# What causes loneliness among household heads: a study based in primary setting in Mumbai, India

**DOI:** 10.1186/s12889-022-13108-w

**Published:** 2022-04-12

**Authors:** Vidya Yadav, Shekhar Chauhan, Ratna Patel

**Affiliations:** 1 Assistant Professor P.G. Department of Geography, College of Commerce, Arts & Science, Patna, Patliputra University, Patna, 800020 India; 2 Department of Family and Generations, International Institute for Population Sciences, Mumbai, India; 3 PhD Research Scholar, Department of Public Health and Mortality Studies, International Institute for Population Sciences, Mumbai, India

**Keywords:** Loneliness, Urbanization, Civic engagement, India

## Abstract

**Background:**

With the pace of urbanization, symptoms of loneliness emerge as one of the most devastating mental illnesses among city dwellers in the modern age. The present study has tried to identify the potential factors and correlates which affect loneliness vulnerability.

**Methods:**

The data for this study were collected from three different areas of Mumbai (i.e., Dadar, Bandra, and Chembur).This study was conducted through a cross-sectional household survey of household heads in the five different housing typologies/ localities between January and June 2016.A total of 450 household data were collected using the quota sampling method. Loneliness was the main dependent variable. The bivariate analysis was used to see the percentage of loneliness among respondents. Bivariate analysis for categorical data was carried out using the chi-square (χ^2^) test. Logistic regression analysis was performed to explore the correlates of loneliness among household heads. The probability of significance was set at 5%.

**Results:**

It was found that around 7 percent of respondents often feel lonely, and 21 percent of respondents sometimes feel lonely in the last seven days preceding the survey date. Household heads with two or more chronic diseases had higher odds (OR = 4.87, CI = 1.52–15.57) of loneliness than household heads without any chronic disease. The odds of loneliness were almost 3 times higher (OR = 3.05; CI = 1.11–8.38) among females as compared to males. Household heads living alone (single) had higher odds (OR = 19.99; CI = 4.14–96.59) to suffer from loneliness than those living in a joint family.

**Conclusion:**

Finding reveals that level of loneliness symptomatology in urban dwellers may be attributed significantly by individual (i.e., morbidity status and sex of respondent), social (i.e., personal relation) and residing locality characteristics. Community psychological intervention along with enhanced civic engagement can reduce level of loneliness in existing slum rehabilitees.

## Background

Urbanization is considered a new avenue of prosperity as it continuously attracts people to a better life. Nevertheless, gradually, it was realized that urbanization is like a two-edged sword [1]; on one side, it provides better opportunities for standard life and health care to the population through better urban services [2]. However, on the other side, the fruits of city life such as privacy and seclusion have led to many physical and psychological problems among people living in urban areas [2]. In this context, it has been anticipated that with the pace of urbanization, symptoms of loneliness emerge as one of the most devastating mental illnesses among city dwellers in the modern age [3]–[5].

Currently, around 30% of the India’s population live in urban areas. It also appraised that by 2025, the share of people living in urban areas is going to be higher by 46% [6]. Previous studies asserted that Indian society passes through the transitional phase of culture [7]. The traditional family system underwent structural and functional changes, which weakened the family ties [8], causing migration and individual disconnection with family. Additionally, hectic city lives leave little time for socializing or leisure living. Therefore, one may find isolation even while living in a big family or a big condominium with several neighbours[9, 10]. Social media has a profound role in compounding the problem by making people connect virtually, diminishing the importance of real face-to-face interaction [11]. This anonymity of living in a big city can end in a scarcity of trust, making it harder to attach. In this way, people are paying a high price for its stretched emphasis on privacy and individuality that many people have no friends and close ones [12]. Also, reaching a threshold point of virtual contacts, isolation backfires probably too when this threshold has been crossed [13]. In this scenario, emotional seclusion may be a tricky thing that strikes different people for different reasons. So, there is no rule or set method to beat loneliness. The lonely person continues to suffer from numerous problems in life.

Empirically loneliness is defined in several ways. It is a natural human feeling triggered by an unpleasant experience of life that occurs when a person's social relationship network is deficient in some significant way, either quantitatively or qualitatively [14]. Sadler (1975) describes “loneliness is caused not by being alone but by being without some needed relationship or set of relationships [15].” He divided loneliness into two types: emotional loneliness: characterized by the lack of an attachment; and social isolation which is manifested through the absence of a social network [15]. Besides, Tiwari (2013) categorized loneliness circumstances into three parts 1) situational loneliness: it arises as a result of adverse socio-economic and cultural situations such as unpleasant experiences due to the disagreement of needs, loss of social contact, migration of distress, interpersonal conflicts, accidents, disasters or emptiness [16]. 2) Developmental loneliness: It appears when individuals are unable to balance, for example, personal inadequacies, separation, poverty, living arrangements, and disability, between innate desire and need [16]. 3) Internal loneliness: it appears to be due to certain inferior personality characteristics such as low self-esteem, feelings of guilt or worthlessness, and poor situational coping skills [16]. In addition, every city dweller values privacy, and therefore the terms of urban living encourage it; hence, loneliness is more often voluntary than imposed. However, when loneliness becomes chronic, it can severely affect both health and well-being; unfortunately, it is continuously emerging as a modern urban life problem [17]. It is a hidden killer that can trigger many problems, including biological dysfunction, psychological distress, and behavioural problems [16, 18]. It is a common stereotype that it mainly affects older people, but previous studies have shown that even younger people suffer from it [19]. Typically, the progression of loneliness has a nonlinear U-shaped distributional curve with people under 25 years of age and people over 65 years of age [20]. Likewise, the magnitude of loneliness shows variations across all ages [21]. In this context, Singh (1991) stated that everyone has to suffer from their ravages at one time or another [22]. Despite this, in India, very little comprehensive research has been done to capture the growing phenomenon of loneliness. However, most related studies concentrated on the elderly population [17].

Thus, there is a greater need for comprehensive studies to fill this research gap, particularly in urban India. Many of these studies lack sound individual loneliness information in an urban locality and data on potential confounders, such as socio-economic status, the role of social capital, and information on substance use. Therefore, this study aimed to examine the pattern and correlates of loneliness among household heads in primary setting in Mumbai, India.

## Methods

### Study site

**Fig.1:**
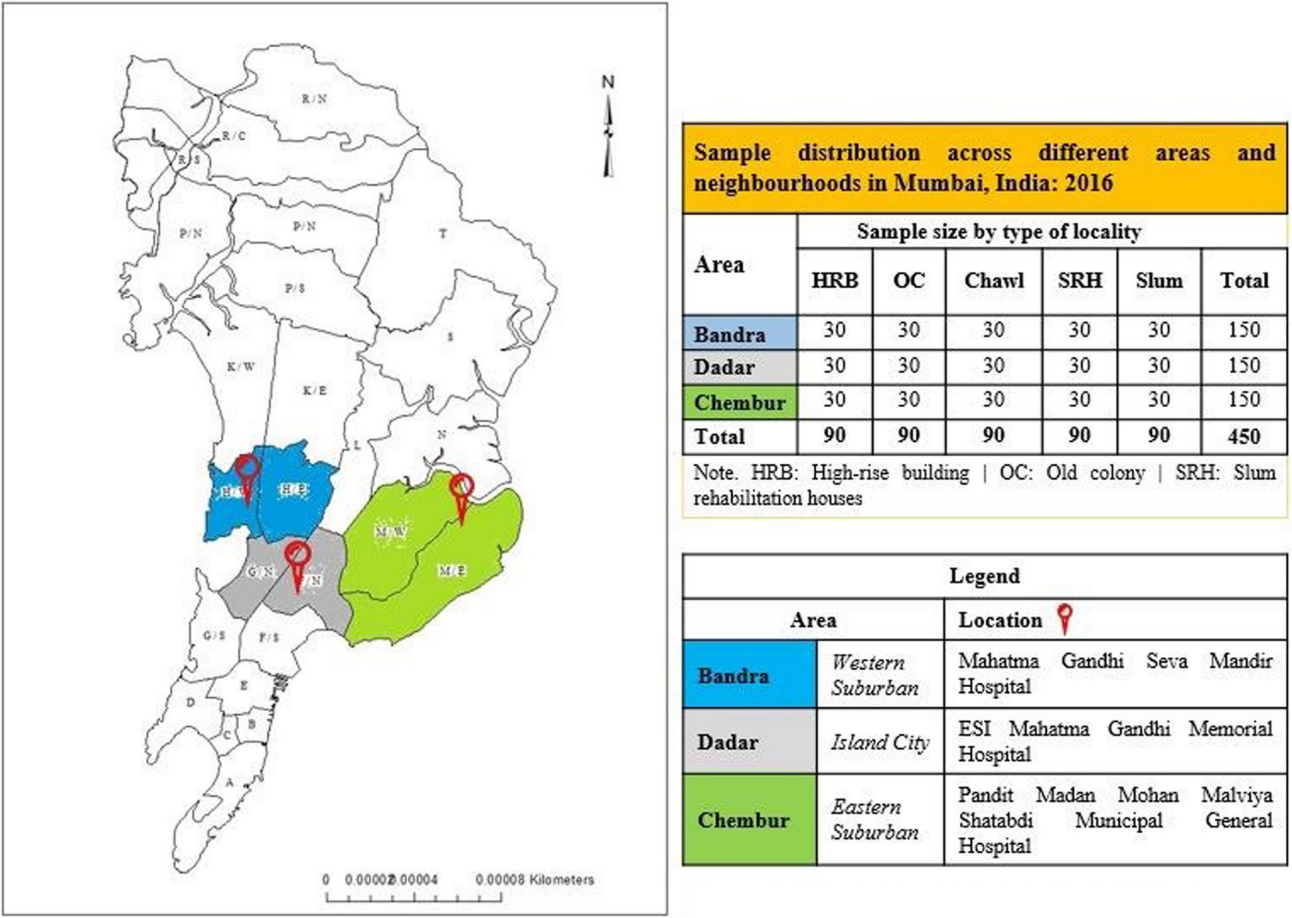
Selected study areas of Mumbai City. Legend: Bandra, Dadar, Chembur

The data for this study were collected from three different areas of Mumbai (i.e., Dadar, Bandra, and Chembur). The selection of these areas was made with the understanding to demarcate the study areas in terms of geographical boundaries [23] because administrative ward boundaries are often redrawn and reshuffled by Brihanmumbai Municipal Corporation (BMC) before the civic election to ensure the desired population size. The selected areas represent different parts of Mumbai, such as Bandra represents the Island city part, whereas Dadar and Chembur, represent a suburban part (refer to Fig. 1).

### Data collection

This study used concurrent mixed method to collect quantitative and qualitative data. The quantitative data were gathered using cross-sectional household survey of household heads in five different housing typologies/ localities between January and June 2016. These localities were High-Rise Residential Buildings (HRB), Old Colonies, Chawls, Slums, and Slum Rehabilitated Houses (SRH). These particular localities were chosen as they are uniformly distributed in selected areas, and they differ in terms of built forms and socio-economic aspects, psychological unity among people living in these housing localities, the feeling of belongingness, or intense use of an area’s commerce, recreational, and learning service [23]. The localities were identified by creating a buffer zone of 5 km aerial radius from a fixed landmark (i.e., main Government Hospital) in selected areas. The selection of a government hospital as a landmark was made in light of its comprehensive serviceability across socio-economic groups in the proximate area. A total of 450 household were interviewed using the quota sampling method: 30 households from each locality of the area were randomly surveyed using a sampling frame. The sampling frame has been created by house listing before the survey. Likewise, each locality consists of 90 sampled households across three areas (i.e., 30 households × 3 areas), and 150 households from each area were taken from five different localities (i.e., 30 households × 5 localities). The purpose of fixing a quota of samples was to avoid high non-response rates, particularly from HRB and Old colonies, and to fulfil the desired sample size.

During the preparation of the sampling frame for this study, inclusion criteria were defined as households must be residing in the area at least for five years preceding the date of the survey. Previous studies show that after residing in a certain area/locality for over a year, residents show adaptability behaviour (i.e., social and health) with prevailing environmental characteristics [24]. The respondents for the study are household heads. In the absence of the household head, the interview was conducted with any other household member aged 18 years or older knowledgeable of the household.

The qualitative data were collected through 5 key informant interview (KIIs), 5 In-depth interview (IDIs) and 5 focused group discussion (FGDs) conducted in each locality. The FGDs were conducted using participants of heterogeneous age group with an assumption to collect diverse viewpoints on the study subject. Further, qualitative data were coded and analysed in Atlas-ti software.

### Ethical approval of studies and Informed consent

The study was conducted with the approval of the Students Research Ethics Committee (SREC) of the International Institute for Population Sciences (IIPS), Mumbai, India. The ethics committee examined the methodological, technical, and ethical soundness of the study. Besides, before conducting the interviews, we obtained the participants' written informed consent and assured them of confidentiality.

### Dependent variable

#### Lonliness

This study uses a direct measure of loneliness adopted from the Longitudinal Ageing Study in India (LASI) survey tool with some modifications [25]. The question was asked, “How often do you feel lonely even after staying with lots of people during the past week?” The response of this question was recorded in three ways (i.e., 0-Hardly ever or never, 1- Some of the time, 2- Often) but, later due to less sample size and study convenience, it was merged and converted into a dichotomous form such as 0- Not lonely (0-hardly ever or never + 1- some of the time), and 1-Lonely (those responded often feel lonely).

### Independent variables

#### Type of locality

Locality typology is based on the built environment of the settlement such as ***Slums:*** Slum refers to the residential areas where dwellings are unfit for human habitation by reasons of dilapidation, overcrowding, faulty arrangements and design of such buildings, narrowness, or faulty arrangement of the street, lack of ventilation, light, or sanitation facilities or any combination of these factors which are detrimental to the safety and health (Census, 2011). ***High-rise residential buildings (HRB):*** High-rise residential buildings refer to tall buildings specially structured for residential purposes. In India, a building higher than 75 feet (23 m), generally 7 to 10 stories, is considered a high-rise. The Municipal Corporation of Greater Mumbai (MCGM) proposed that any building with at least a height of 30 m or nine floors can be categorized as high rise. It is a multi-dwelling unit that may be owned or rented. For this study, we considered those buildings having a minimum of nine-storey and maximum is having no limit. The dwelling unit here is considered as one unit of the flat of the building. ***Chawls:*** A Chawl is a building form particularly found in Mumbai. They are often one or two and sometimes three storeys with about 10 to 20 tenements, referred to as kholis, which means room on each floor. A usual tenement in a chawl consists of one all-purpose room that functions as a living and sleeping room. Families on each floor have or may not have to share a common block of toilets. ***Old colonies:*** Here, old colonies refer to those colonies established a long time ago by the migrants of the same ethnic or cultural group. In this study, we have selected Parsis (migrated from Arab to India) and Sindhi colony, as they are considered as one of the oldest settlers of Mumbai. ***Slum Rehabilitated House (SRH):*** The Government of Maharashtra has launched a comprehensive slum rehabilitation scheme by introducing an innovative concept of using land as a resource and allowing incentives on floor space index (FSI) in the form of tenements for sale in the open market, for cross-subsidization of the slum rehabilitation tenements which are to be provided free to the slum-dwellers.

#### Social capital

Present study adopted standard set of multidimensional indicators on social capital used in previous studies with some modification [26, 27]. The gathered information on social capital later categorised into its different dimensions such as personal relation, social network and support, civic engagement, and trust and cooperative norms. The cronbach’s alpha (α) test was performed for each dimensional index to check its reliability. The study accepted reliability greater than 0.5 α value [28–30]. The detail description of question associated with indexes are as follows: *(i) Personal Relationships*: this index developed bases on the following questions 1. Do you have a close connection with your relative members? 2. How often do you meet with your relatives? 3. How well you know your friends? 4. How often do you meet with your friends? 5. Whether do you have close friends? 6. Do you satisfy with your family life? The cronbach’s alpha (α) value was found to be 0.68, with a standardized alpha (α) value of 0.67.

*(ii) Social network & support: This index was developed based on the following questions* 1. Do you or family member feels supported/helped by relatives? 2. Would you like to go more often to your relatives? 3. In an urgent situation, what do you think your friends/ neighbour will help you? 4. Whether within one year you and your family member has given any advice/ suggestion? 5. Have you done unspoken help for your friends 6. On whom you rely most for financial assistance? The cronbach’s alpha (α) value was found to be 0.77, with a standardized alpha (α) value of 0.77.

*(iii) Civic engagement:* this index developed based on following questions 1. Whether you or your locality members ever tried to maintain any social order in locality2. In the past year, whether this community member had tried to solve a problem? 3. Have you demanded anything from your electoral leaders? 4. Do you a member of any socio-political organisation? 5. Do you participate in any socio-political gathering? The Cronbach’s alpha (α) value was found to be 0.62, with a standardized alpha (α) value of 0.62.

*(iv) Trust and cooperative norms:*1. Whether your friends/ neighbour is trustworthy and reliable? 2. Do you feel a sense of community? 3. Do you feel safe in this locality 4. Whether female feel safe in night in this locality? The Cronbach’s alpha (α) value was found to be 0.53, with a standardized alpha (α) value of 0.54.

Other selected covariates are Chronic disease status: it includes the type of chronic morbidity and number of days/years of illness and its treatment. Furthermore, frequency and reason behind Substance abuse has been captured. Other than that, availability and accessibility to space for physical activity variables has also been analysed. Apart from all these, Age of the household head, Sex, Per capita Income, Years of schooling of HH, Family type, Marital status, and working status been included.

### Data Analysis

The obtained data from the survey were processed (i.e., entry & editing) with the help of CS-pro 6.2 software, later cleaned data were analysed using STATA -13.1 Package. The bivariate analysis was used to see the percentage of loneliness among respondents. Bivariate analysis for categorical data was carried out using the chi-square (χ^2^) test. Three models of logistic regression were constructed to assess the correlates of loneliness among household heads. The probability of significance was set at 5%. The conceptual thinking behind these models were to understand the built form of a neighbourhood in an aesthetic form that influences the psychology of its residents. In some places where the built form is unfavourable, residents face a variety of physical and physiological challenges. Individual and social factors assist residents in coping in a negative or unfavourable built environment. So, in this direction the Unadjusted Model-1 initiated to examined the relationship between the types of localities and loneliness. Model-2 assessed the association of social capital (i.e., personal relationship, civic engagement, trust, and cooperation) with loneliness. Model-3 was finally adjusted with all the confounding variables.

## Results

### Household head characteristic

The demographic characteristics of the household head living across localities are shown in Table 1. It was found that the distribution of household heads by age group was inconsistent across localities. In general, most household heads (39%) were young age group between 25–45 years. Slum followed HRB comprises the highest percentage of 25–45 years household head population whereas old colonies followed by HRB have a higher level of older household head population. The sex-wise distribution of household heads is significantly skewed towards male counterparts compare to females in general. However, slum followed by HRB consists the highest number of the female household head by 18 percent and 11 percent, respectively. Four-fifth (82%) of the respondents were currently married, followed by others (widow/widower), 15 percent, and 3 percent never married. Besides, it was found that people living in old colonies had a high mean level of years of schooling, per capita income, and years of stay, followed by HRB.

**Table 1:** Percentage distribution of respondents in selected localities by background characteristics, Mumbai, India: 2016

Background Characteristics	Type of Localities					
	**HRB**	**Slums**	**SRH**	**Chawls**	**OC**	**Overall**
**Age-group (years)**	**N (%)**	**N (%)**	**N (%)**	**N (%)**	**N (%)**	**N (%)**
25–45	39 (43.3)	56 (62.2)	38 (42.2)	38 (42.2)	3 (3.3)	**174 (38.6)**
45–65	17 (18.9)	33 (36.7)	42 (46.7)	48 (53.3)	23 (25.6)	**163 (36.2)**
≥ 65	34 (37.8)	1 (1.1)	10 (11.1)	4 (4.4)	64 (71.1)	**113 (25.1)**
**Sex**						
Male	80 (88.9)	74 (82.2)	80 (88.9)	82 (91.1)	81 (90.0)	**397 (88.2)**
Female	10 (11.1)	16 (17.8)	10 (11.1)	8 (8.9)	9 (10.0)	**53 (11.8)**
**Marital status**						
Never married	1 (1.1)	NA	NA	5 (5.6)	9 (10.0)	**15 (3.3)**
Currently married	74 (82.2)	76 (84.4)	81 (90.0)	77 (85.6)	61 (67.8)	**369 (82.0)**
Others	15 (16.7)	14 (15.6)	9 (10.0)	8 (8.9)	20 (22.2)	**66 (14.7)**
**Total (N)**	90 (100)	90 (100)	90 (100)	90 (100)	90 (100)	**450 (100)**
Note. *HRB*: High-rise building | *OC*: Old colony | *SRH*: Slum rehabilitation houses						

Table 2 depicts the mean distribution of respondent in selected localities by background characteristics. Mean years of schooling was highest in Old colonies (17.2 years) and lowest among respondents residing in slums (7 years).

**Table 2:** Mean distribution of respondents in selected localities by background characteristics, Mumbai India: 2016

	**Mean**	**[SD]**	**Mean**	**[SD]**	**Mean**	**[SD]**	**Mean**	**[SD]**	**Mean**	**[SD]**	**Mean**	**[SD]**
	HRB		Slums		SRH		Chawls		OC		**Overall**	
Years of schooling	16.1	[2.2]	7.0	[3.8]	8.4	[4.3]	11.4	[3.5]	17.2	[2.3]	**12.0**	**[5.2]**
Per Capita Income (in '000)	490	[345]	84	[61]	73	[37]	150	[113]	561	[514]	**272**	**[352]**
Length of stay (in Years)	32.7	[20.6]	19.3	[12.3]	31.5	[15.3]	27.7	[19.0]	53.4	[20.6]	**32.9**	**[21.0]**
**Total (N)**	**90**		**90**		**90**		**90**		**90**		**450**	
Note. *HRB*: High-rise building | *OC*: Old colony | *SRH*: Slum rehabilitation houses												

### Level of loneliness in the study area

**Fig.2:**
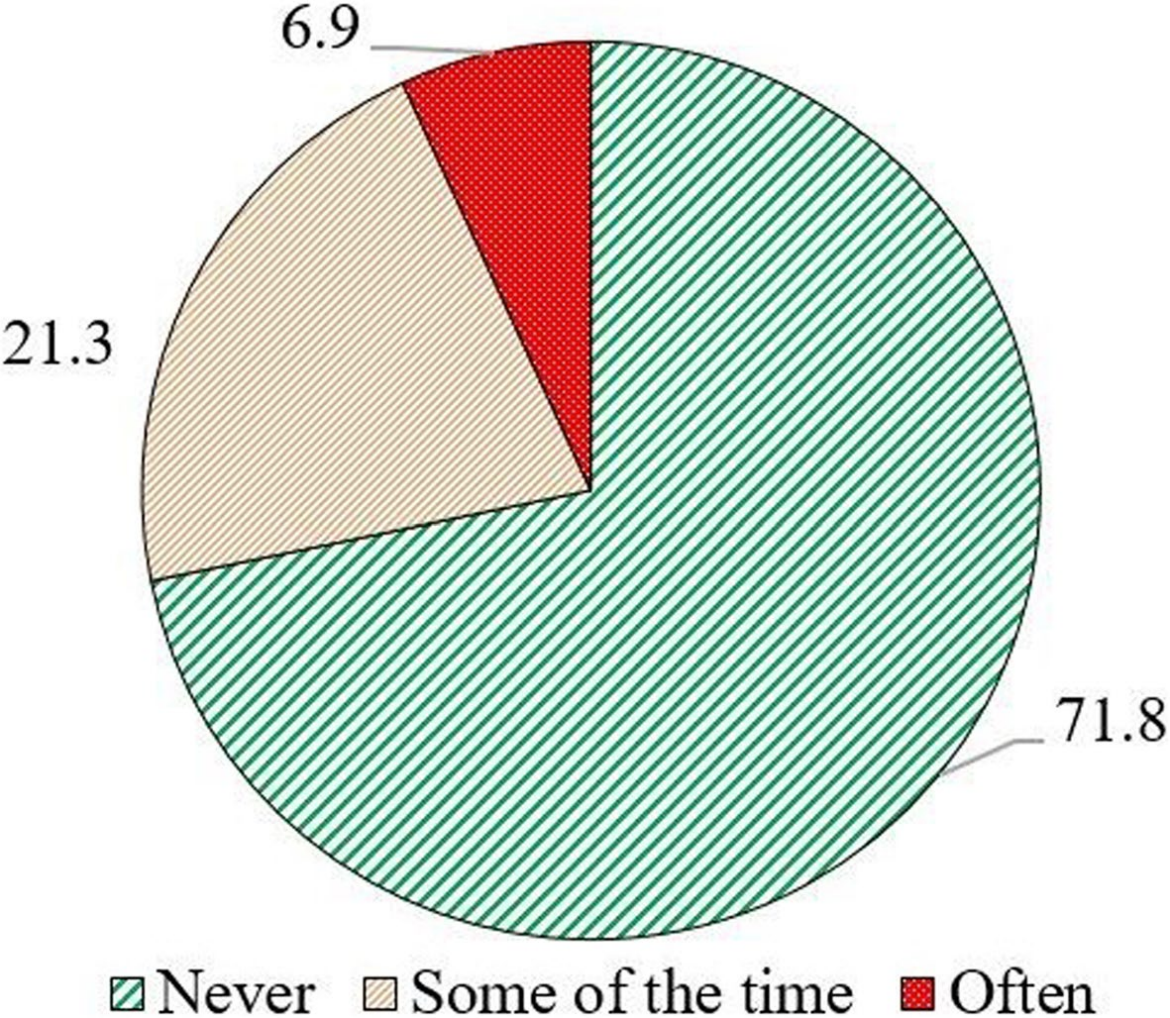
Percent distribution of household heads who felt lonely in Mumbai. Legend:Some of the time, Often

Figure 2 shows the percent distribution of household heads who felt loneliness in last seven days categorized as never, Some of the time, and Often. For further analysis (logistic regression analysis), responses were converted into a dichotomous form such as never lonely and lonely.

### Distribution of lonely people by their background characteristics

Table 3 shows the level of loneliness pattern among household heads by their background characteristics. The level of loneliness was varying with the type of locality, among the household heads of the SRH, 60% reported about loneliness followed by old colonies (33%). The chi-square value (χ^2^—66.3; *P* = 0.000) is showing a significant association of loneliness with locality type. Loneliness was widely prevalent across all the age group of household heads but it was considerably high among 65 years and above aged, among them 40 percent affected with loneliness. “*I am old now and retired long back, lost my wife 3 years back, losses and lack in all my skill and abilities, I feel lonely now and not interested in anything (Male, 85 years, Old colony, Dadar).*

**Table 3:** Percent distribution of household heads who reported loneliness by different background characteristics, Mumbai India 2016

		**Never**	**lonely**	
**Age of the household head**	25-45 years	82.2	17.8	Χ^2^ = 20.758, Pr = 0.001
	45-65 years	70.6	29.5	
	65 and above	57.5	42.5	
**Sex**	male	77.1	22.9	Χ^2^ = 46.746, Pr = 0.001
	female	32.1	67.9	
**Year of schooling**	More than 12	81.6	18.4	Χ^2^ = 17.272, Pr = 0.001
	Up to 12	63.9	36.1	
**Family type**	Joint	63.3	36.7	Χ^2^ = 61.602, Pr = 0.001
	Nuclear Plus	83.0	17.0	
	Nuclear	78.6	21.4	
	Single	17.7	82.4	
**Marital status**	Currently married	79.4	20.6	Χ^2^ = 58.852, Pr = 0.001
	Others	37.0	63.0	
**Personal relation**	Low	73.0	27.0	Χ^2^ = 0.605, Pr = 0.436
	High	69.6	30.4	
**Family support**	Low	73.4	26.6	Χ^2^ = 0.861, Pr = 0.353
	High	69.4	30.6	
**Trust and cooperation**	Low	64.0	36.0	Χ^2^ = 16.797, Pr = 0.001
	High	81.5	18.5	
**Civic engagement**	Low	60.2	39.8	Χ^2^ = 19.963, Pr = 0.001
	High	79.6	20.5	
**Smoking**	No	69.5	30.5	Χ^2^ = 13.825, Pr = 0.001
	Frequently everyday	62.9	37.1	
	everyday	92.6	7.4	
**Alcohol abuse**	No	69.1	30.9	Χ^2^ = 12.814, Pr = 0.002
	Weekly	69.8	30.2	
	Occasionally	93.8	6.3	
**Physical activity**	Inactive	75.2	24.8	Χ^2^ = 3.365, Pr = 0.067
	Active	67.4	32.7	
**Chronic disease status**	No disease	79.6	20.4	Χ^2^ = 34.803, Pr = 0.001
	One chronic disease	67.5	32.5	
	Two/More chronic disease	40.4	59.6	
**Type of locality**	HRB	85.6	14.4	Χ^2^ = 66.324, Pr = 0.001
	Slums	83.3	16.7	
	Chawls	83.3	16.7	
	Old colonies	66.7	33.3	
	SRH	40.0	60.0	

Chronic illnesses show positive relationship with loneliness. The level of loneliness increases three folds among those household heads who have two or more chronic diseases compared to no chronic diseased household head. The prevalence of loneliness among females (68%) was observed nearly three times more than males (23%). The loneliness was reported high among the adults living single. Compared to currently married household head (21%), the prevalence of loneliness was three times higher among household heads (63%) with other marital status. “*As I am Parsi by religion, and in our religion inter-caste marriage is restricted. I was in love with a boy with other religion, my parents didn’t agree with the relationship and I decided to remain unmarried. Since then, I am single, my parents are no more now and I feel very lonely, like no motivation in my life” (Female, 56 years, Old colony, Bandra).*

Those who smokes 'frequently everyday' among them, 37 percent have reported loneliness. One-third of physically active household head suffer with loneliness, this loneliness level is 8 per cent higher than those who were physically inactive but feel lonely. Household heads with high personal relationships and family support may see a higher level of loneliness than their counterpart. “*My whole life I had devoted to my relatives, invested money in education and marriages, but in return I got nothing except tension, stress and lost my self-respect too. Now, I feel it would have better if I have not wasted time in all these and remain happy in my small family. (Male, 62 years, Chawls, Chembur).* Besides, the household head having low trust in relationships and less cooperative, perceived more loneliness. Nevertheless, respondents with low civic engagement resulted in higher loneliness.

### Correlates of loneliness

In this study, three different logistic regression models (refer to Table 4) were used to understand the correlates of loneliness.

**Table 4:** Adjusted odds ratio of loneliness by selected background characteristics, Mumbai India 2016

			**Model 2**		**Model 3**	
	**OR**	**SE**	**OR**	**SE**	**OR**	
**Sex**						
Male®						
Female					3.05**	[1.11 8.38]
**Marital status**						
Currently married®						
Others					3.83***	[1.47 9.94]
**Family type**						
Joint®						
Nuclear Plus					2.25	[0.53 9.58]
Nuclear					3.05**	[1.16 8.03]
Single					19.99***	[4.14 96.59]
**Year of schooling**						
More than 12 years®						
Up to 12 years					2.44*	[0.89 6.65]
**Personal relation**						
Low®						
High			2.26***	[1.27 4.00]	2.57**	[1.11 5.94]
**Civic engagement**						
Low®						
High			0.37***	[0.24 0.58]	0.30***	[0.14 0.65]
**Trust and cooperation**						
Low®						
High			0.28***	[0.17 0.48]	0.19***	[0.09 0.41]
**Chronic disease status**						
No chronic disease®						
One chronic disease					1.09	[0.50 2.37]
Two /More chronic disease					4.87***	[1.52 15.57]
**Alcohol use**						
No®						
Weekly					3.88**	[1.05 14.33]
Occasionally					0.57	[0.11 3.06]
**Type of locality**						
HRB®						
Slums	1.18	[0.53 2.66]			3.52	[0.46 27.08]
SRH	8.88***	[4.31 18.31]			51.0***	[6.84 380.39]
Chawls	1.18	[0.53 2.66]			3.7	[0.62 22.22]
Old colonies	2.96***	[1.42 6.16]			3.4*	[0.82 14.08]
**_cons**	0.17	[0.09 0.30]	0.73*	[0.51 1.05]	0.01***	[0.00 0.08]
**Log-likelihood**	-236.12		-244.12		-147.71	
Note: ®- Reference Category; *p < .05**p < .01; ***p < .001; Other controlled confounding variables in Model—3: Age, PCI, Family support, Smoking, Physical activity						

Unadjusted odds ratio of Model-1 shows that household heads in slum rehabilitated houses (OR = 8.88; CI = 4.31–18.31) and old colonies (OR = 2.96; CI = 1.42–6.16) had higher odds of loneliness than the referenced category, but, after adjustment of other covariates, the odds of loneliness were much higher (refer Model-3). Unadjusted Model-2 shows that the odds of loneliness were higher (OR = 2.26, CI = 1.27–4.0) among household heads with high personal relation as compared to their counterparts who had low personal relations. The high level of civic engagement, and trust and cooperation significantly reduce the loneliness among the household head by 63% and 72%, respectively, in Model-2.

In Model 3, household heads with two or more chronic diseases had higher odds (OR = 4.87, CI = 1.52–15.57) of loneliness than household heads without any chronic disease. The odds of loneliness were almost 3 times higher (OR = 3.05; CI = 1.11–8.38) among females as compared to males. Household heads living alone (single) had higher odds (OR = 19.99; CI = 4.14–96.59) to suffer from loneliness than those living in a joint family. Years of schooling show a significant relationship with loneliness; the household heads having up to 12 years of schooling had higher odds (OR = 2.44; CI = 0.89–6.65) of loneliness compared to the reference category.

## Discussion

Loneliness is a growing health epidemic, particularly among city dwellers due to changed lifestyles and social adversities. The present study reinforced and extends the previous findings on loneliness, particularly in city dwellers of India. The salient findings are: first, the dwellers of the SRH locality perceived a comparatively higher level of loneliness than other localities. Second, those household heads who suffer from multiple morbidities also suffer from loneliness. Third, the prevalence of loneliness was more prominent among females than in males. Fourth, those household heads having a high level of personal relationships have more likelihood to suffer from loneliness. Fifth, civic engagement, and trust and cooperation show a negative association with loneliness. The data used for this study was collected five years ago, and given the current pandemic situation, how reliable and useful are the estimates are worth probing? Despite data being a bit old, the comprehensiveness and usefulness of the study findings cannot be ruled out completely. However, it is true that with the advent of coronavirus, the study findings may not hold by large in the current context. As this study explored the prevalence and correlates of loneliness among household heads, it is expected that the prevalence of loneliness might have increased after coronavirus. On a similar note, the factors linked to loneliness might have been not the same due to coronavirus. Accordingly, a new study exploring loneliness among household heads in the same study area may provide better estimates.

The study finds that more than one-fourth (28%) of people in Mumbai suffer from loneliness. However, its percentage was varying by locality type. The unadjusted and adjusted OR value shows that SRH followed by old colony residents strongly associate with loneliness. Previous studies also asserted that SRH residents complain about the unfavourable built environment, which reduces the sensory connectedness and restricts traditional flows with other neighbours due to random allocation of flats [31]. It eventually hampers their collective identity, weakened their social support network, and significantly leads to loneliness. Hence, they want to go back to the horizontal slum again; this willingness considers as a rebound phenomenon [32]. On the other hand, old colonies comprised mainly of old-age Parsi and Sindhi populations. The present study asserted that factors like disability and health, loss of a spouse, living alone, and ageing were significantly associated with their perceived loneliness. There is no direct empirical evidence on this issue. However, some previous studies indicate about declining demographic trend of these communities, particularly Parsis. Those studies gave reasoning such as never married, late marriage, and migration about their declining population, etc. [33].

Physical multimorbidity (≥ 2 physical diseases) was associated with increased odds for loneliness. It might be happening due to a greater number of chronic diseases creating hindrance to engage in social activities and roles such as spouse, parent, and worker. This finding is consistent with the previous studies [34, 35]. Multimorbidity leads to social isolation which can be a cause of loneliness [36]. Furthermore, Barlow et al. (2015) demonstrated that multimorbidity affects physical functioning, which can further affect loneliness [37]. Another plausible explanation was provided by Kristensen et al. (2019), where they associated loneliness with quality of the relationships and not with the quantity of the relationships [34]. They suggested that multimorbidity may affect loneliness by reducing the quality in relationship [34]. Furthermore, Jessen et al. (2018) also provided a plausible explanation for the relationship between multimorbidity and loneliness and stated that household head with multimorbidity have to deal with health care system on a regular basis, thereby restricting their social participation leading to loneliness [38]. To add more, people with multimorbidity may have to leave labour market thereby reducing the everyday contact with colleagues, which could also be a plausible factor of loneliness among them [38].

Gender is an important determinant in the context of perceived loneliness; and in agreement with previous research results indicate that females were lonelier than their male counterpart [39, 40]. In contrast, few studies have also noted an otherwise outcome where men were more likely to experience loneliness than their female counterparts [41, 42].The likelihood of odds was also significantly three times more among females after adjusting all other factors. This situation occurs because females are not highly interconnected or have cohesive sets of friends compared to their counterparts. Moreover, women are also more likely to precise and share their emotions with, and be more responsive to the emotions of others [43]. However, the stigma related to loneliness, mainly among men, is different; men are less likely to interact and intimate closure than women; but relational connectedness is more important for ladies than men [44].

In concordant with previous studies[45, 46], this study noticed higher odds of loneliness among older adults who belonged to other category of marital status than the older adults who were married. In a marital union, protection from social loneliness come from involvement with spouse, thereby reducing loneliness [45]. Women not in marital union may perceive loneliness due to the adjustment in a new role of singlehood [46]. Another plausible explanation suggest that with increasing age, friendship ties become weak and the spouse is the only person with whom older adult feel attached; thereby reducing loneliness among them [47].

Previous studies found an inverse relationship between social capital with loneliness and asserted that strong relations with relatives and friends lower stress [48]. Although different dimensions of social capital attribute, unlike personal characteristics. Results show that a high level of personal relationships leads to a high level of loneliness. This positive, strong relationship considers as a dark side of close personal relation [49]. Fafchamps (2006) argues that sometimes an excessive amount of social bonding and connection results in pressure and tension, and it becomes negative, creating conformity instead of variety[50]. In contrast, a high level of trust and cooperation and civic engagement reduces loneliness by three quarters. It is evident that the quality of social engagement and trust is protective against loneliness and a key factor to achieve personal and social desired goals [9, 20].

The findings of this study shall be interpreted in line of some noteworthy limitations. The study acknowledges that the physical illness was self-reported and was not verified against other data sources such as medical records. This might have resulted in misreporting in some instances, especially as evidence suggests that while self-reports of physical illness mostly accord with doctor’s reports, not all diseases are reported with equal accuracy, and there might also be age-related differences in the reliability of reports for some diseases. Nonetheless, the cross-sectional nature of the present study restricts the claims of causal inferences; hence we cannot conclude that an increase in loneliness is caused by any particular factor or vice-versa. As this study was cross-sectional, we could not establish causality or determine the direction of the observed association. Also, the information on loneliness was self-reported and was assessed using one item only. Furthermore, the study findings shall not be generalized to a larger set of population as purposive sampling was done in a metropolitan region only.

## Conclusion

It has long been noted that life style of urban dwellers adversely affects their health not only physically but mentally too. In this context, loneliness rapidly emerged as a public health concerns in urban part. Hence, current study tried to examined the level of loneliness and its significant factors. Finding reveals that level of loneliness symptomatology in urban dwellers may be attributed significantly by individual (i.e., morbidity status and sex of respondent), social (i.e., personal relation) and residing locality characteristics. On the other hand, some factors (i.e., civic engagement, and trust and cooperation) identified as inversely associated with loneliness. In nutshell, study concludes that that there is a significant effect of locality in the subjective feeling of loneliness. Slum rehabilitees were found to be significantly lonelier than their counterpart localities. Furthermore, many identified the risk factors of loneliness could be avoided if timely preventive measures could be taken along with adequate care. Improvement in aesthetic design of future slum rehabilitation projects can promote socially cohesive environment.

## Data Availability

All relevant data are within the paper.

## Abbreviations

**BMC**: Brihanmumbai Municipal Corporation
**HRB**: High-Rise Residential Buildings
**IIPS**: International Institute for Population Sciences
**LASI**: Longitudinal Ageing Study in India
**MCGM**: Municipal Corporation of Greater Mumbai
**SRH**: Slum Rehabilitated Houses
